# Failure of Intravenous or Intracardiac Delivery of Mesenchymal Stromal Cells to Improve Outcomes after Focal Traumatic Brain Injury in the Female Rat

**DOI:** 10.1371/journal.pone.0126551

**Published:** 2015-05-06

**Authors:** L. Christine Turtzo, Matthew D. Budde, Dana D. Dean, Eric M. Gold, Bobbi K. Lewis, Lindsay Janes, Jacob Lescher, Tiziana Coppola, Angela Yarnell, Neil E. Grunberg, Joseph A. Frank

**Affiliations:** 1 Center for Neuroscience and Regenerative Medicine, Uniformed Services University of the Health Sciences, Bethesda, Maryland, United States of America; 2 Frank Laboratory, National Institutes of Health, Bethesda, Maryland, United States of America; 3 Department of Medical and Clinical Psychology, Uniformed Services University of the Health Sciences, Bethesda, Maryland, United States of America; 4 National Institute of Biomedical Imaging and Bioengineering, National Institutes of Health, Bethesda, Maryland, United States of America; Rutgers—New Jersey Medical School, UNITED STATES

## Abstract

Mesenchymal stromal cells secrete a variety of anti-inflammatory factors and may provide a regenerative medicine option for the treatment of traumatic brain injury. The present study investigates the efficacy of multiple intravenous or intracardiac administrations of rat mesenchymal stromal cells or human mesenchymal stromal cells in female rats after controlled cortical impact by *in vivo* MRI, neurobehavior, and histopathology evaluation. Neither intravenous nor intracardiac administration of mesenchymal stromal cells derived from either rats or humans improved MRI measures of lesion volume or neurobehavioral outcome compared to saline treatment. Few mesenchymal stromal cells (<0.0005% of injected dose) were found within 3 days of last dosage at the site of injury after either delivery route, with no mesenchymal stromal cells being detectable in brain at 30 or 56 days post-injury. These findings suggest that non-autologous mesenchymal stromal cells therapy via intravenous or intracardiac administration is not a promising treatment after focal contusion traumatic brain injury in this female rodent model.

## Introduction

In the U.S. in 2010, 2.5 million people had traumatic brain injury (TBI) of enough severity to seek care at a hospital [1]. TBI-related death rates in men were 25.4 per 100,000 US population versus 9.0 per 100,000 US population in women [2]. Since these data [1, 2] only include cases involving individuals who sought medical care at a hospital, the incidence of TBI including mild cases is likely even higher. Aside from supportive care measures, there are no currently available pharmacological treatments for traumatic brain injury [3].

Mesenchymal stromal cells (MSC), also known as mesenchymal stem cells, have been proposed as a possible therapy for a variety of neurological disorders, including TBI [4]. Known to secrete cytokines and growth factors, MSC may act in a paracrine fashion to limit the extent of injury or to facilitate repair of damaged tissues [5]. Initial reports of MSC in rodent models of TBI reported that after a single intravenous or intra-arterial injection, MSC homed to the site of injury and resulted in long-term improvements in neurobehavioral function [6–8].

The purpose of the present study was to determine the optimal route of peripheral vascular delivery and whether multiple doses can maximize transplanted cell survival and functional recovery in a rat TBI model of focal contusion. We also investigated whether administration of MSC labeled with superparamagnetic iron oxide nanoparticles (SPION) would permit *in vivo* and *ex vivo* MRI tracking of labeled stem cells over a time course after the injury.

## Materials and Methods

### Animals

All studies were performed according to the National Research Council’s *Guide for the Care and Use of Laboratory Animals*. The protocol was approved by the National Institutes of Health Clinical Center Animal Care and Use Committee (protocol numbers LDRR 09–03 and LDRR 12–02). All surgery was performed under isoflurane anesthesia, and all efforts were made to minimize suffering through the administration of acetaminophen post-injury as detailed in the section describing the injury protocol below.

Female Wistar rats (n = 177) of initial age 8–12 weeks and weight of 200–250 g were obtained from Charles River Laboratories (Wilmington, MA). Distribution of female rats for long-term neurobehavioral and MRI studies was as follows: 34 rats for rat MSC (rMSC) versus saline intravenous (IV) cohort; 24 rats for rMSC versus saline intracardiac (IC) cohort; 24 rats for human MSC (hMSC) versus saline IV cohort; 28 rats for hMSC versus saline IC cohort; 20 rats for uninjured neurobehavioral controls; 7 rats for uninjured MRI controls. For short-term experiments, 40 additional rats were used to determine if transplanted stem cells were detectable by histology in injured brain within the first 10 days after injury. For rat bone marrow MSC isolation, male Wistar rats (n = 20) of initial age 6–12 weeks and weight of 200–300 g were obtained from Charles River Laboratories (Wilmington, MA). Rats were pair-housed in temperature-controlled conditions under 12 hour light/dark cycles, and had access to standard laboratory rodent chow and water *ad libitum*.

### Controlled Cortical Impact (CCI) Injury

The rat CCI model was performed as previously described [9] using an electromagnetic device (Impact One stereotaxic impactor, my NeuroLab.com/Leica Microsystems, Richmond, IL) with a 5 mm flat impactor tip over the left motor cortex (2.5 mm left lateral and 1.0 mm anterior of Bregma) at a velocity of 5 m/s, depth of 2.0 mm, and dwell time of 100 msec. For each cohort of rats, the same surgeon performed all CCI surgeries. After injury, animals were observed continuously until they recovered from anesthesia and were able to move freely about their recovery cages. Rats were subsequently observed a minimum of two times a day, and more frequently on days when MRI, neurobehavioral testing, or administration of stem cell versus saline doses occurred. For post-injury analgesia, acetaminophen (100 mg/kg) was administered twice daily by mouth for the first 48 hours after injury.

### MSC Preparation and Characterization

Rat MSC were isolated aseptically from bone marrow derived from the femurs of 6 to 10 week old male Wistar rats and characterized by flow cytometry analysis for selected surface markers per protocol as previously described [10, 11]. rMSC cell migration was assessed using the QCM Chemotaxis Cell Migration Assay (8 μM, EMD Millipore, Billerica, MA) per manufacturer’s instructions. Monocyte chemoattractant protein-1 (MCP-1), stromal cell-derived factor 1 (SDF-1), platelet-derived growth factor-AA (PDGF-AA), and platelet-derived growth factor-BB (PDGF-BB) used to assess cell migration was obtained from Peprotech (Rocky Hill, NJ). rMSC at passage 2 to 4 were administered for transplantation.

Human MSC were derived and characterized by the NIH Bone Marrow Stromal Cell Transplantation Center [12, 13] and cultured as previously described [14]. Superparamagnetic iron oxide nanoparticles (SPION) (ferumoxides (Feridex, Bayer AG, Germany) were complexed to the clinical transfection agent protamine sulfate as previously described [15]. hMSC at passage number less than 5 were magnetically labeled by co-culture with ferumoxides-protamine sulfate (Fe-Pro) in fresh media for 2 hours at 37°C as described [16].

### Experimental cohorts

**Fig 1:**
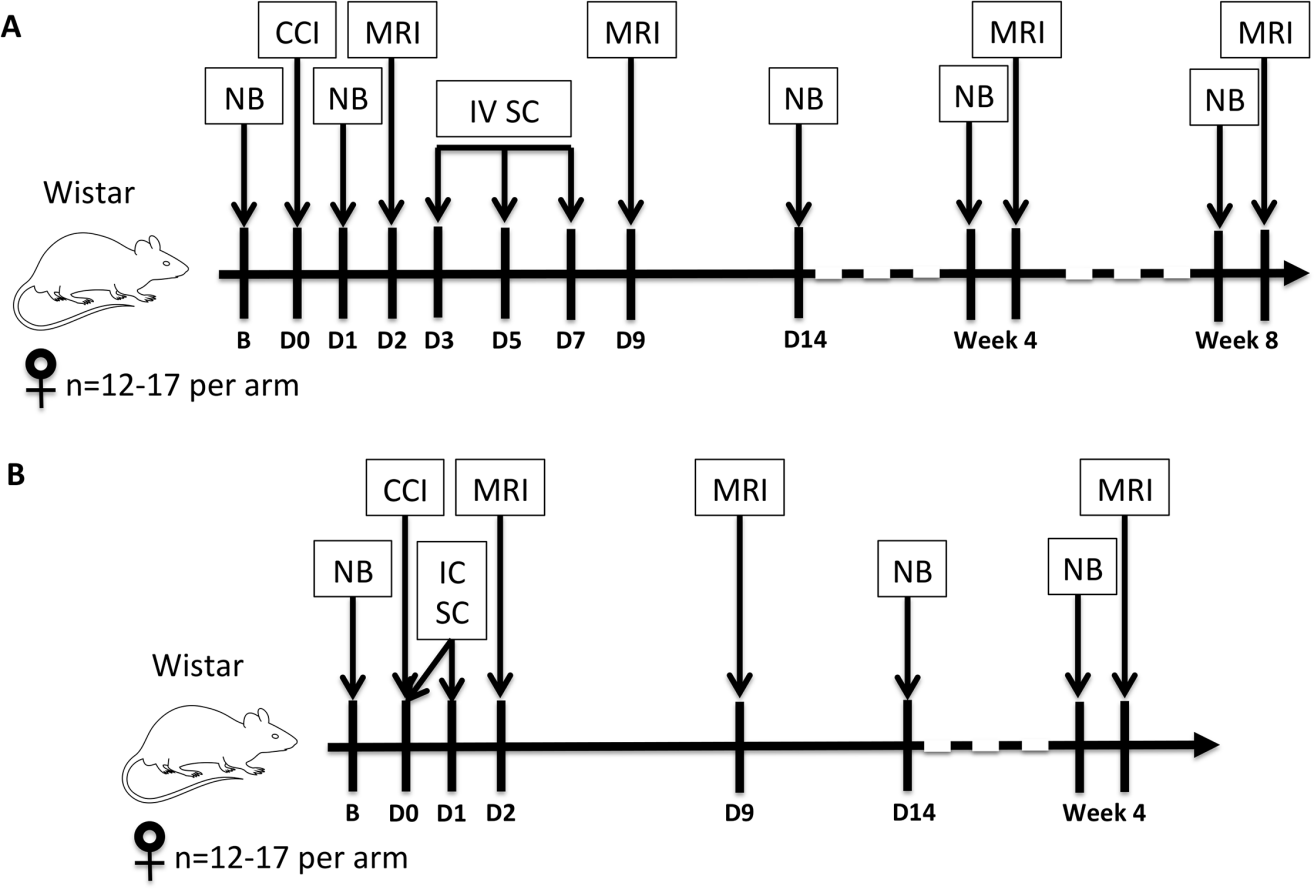
Experimental design to investigate the efficacy of stem cell administration after focal TBI. A) Schematic for cohorts in which rat (rMSC) or human (hMSC) mesenchymal stromal cells were administered intravenously (IV) on days 3, 5, and 7 after controlled cortical impact (CCI); B) Schematic for cohorts receiving intracardiac (IC) injections of stem cells on days 0 and 1 post-CCI. B = baseline; D = day post-injury; NB = neurobehavioral testing; IV SC = intravenous stem cell injection; IC SC = intracardiac stem cell injection.

Fig 1 depicts an overview of the experimental timelines for the IV and IC arms of the study. Prior to IV stem cell versus placebo treatment, each rat’s initial post-injury MRI (Day 2) was assessed, and Day 1 neurobehavioral scores were assessed. Similar appearing lesions and neurobehavioral scores were paired, then rats of each pair were randomized into treatment versus saline groups. This was done to ensure that the overall burden of injury by MRI and neurobehavioral testing was equivalent in both IV treatment groups prior to stem cell versus saline treatment.

For IV treatments, animals were anesthetized with isoflurane and temporary tail vein catheters were placed for ease of administration. To trigger vasodilation to reduce stem cell trapping within the lung vasculature, all animals in the IV treatment cohort (both saline and stem cell) were treated with nitroprusside at a dose of 25 μg from a 1 mg/mL solution followed by 100 μL saline [17] 5 minutes prior to the injection of MSC. On days 3, 5, and 7 post-injury, 5 million rat or human MSC in 200 μL sterile heparinized phosphate buffered saline (PBS) or 200 μL sterile heparinized PBS (vehicle group) were administered via tail vein catheter, followed by a flush of heparinized normal saline.

For the IC cohorts, after anesthesia with isoflurane rats received an analgesic dose of buprenorphine (0.03 mg/kg subcutaneously) prior to preparation of the left chest wall and administration of local anesthesia with bupivacaine 0.5% (0.05 mL intradermally). IC injections were performed under ultrasound guidance with an Acuson Sequoia (8–15 MHz) ultrasound unit (Siemens, Malvern, PA). One million MSC in a volume of 200 μL sterile PBS or 200 μL sterile PBS alone were slowly administered into the left ventricle of the heart over a one minute period of time. MRI was not possible immediately post CCI in the IC cohort of rats because the initial injections were completed within 15 minutes of injury.

### MRI

*In vivo* MRI of the brain was performed on days 2, 9, and 30 post-CCI on a 7 T Bruker Biospec (Billerica, MA) using a 40 mm inner diameter quadrature volume coil (Doty Scientific, Columbia, SC) as previously described [9]. *In vivo* MRI of healthy control age-matched female rats (n = 7) was used to determine the variability in brain volumes. *Ex vivo* MRI was performed on paraformaldehyde-fixed brains on a 7 T vertical bore magnet (Bruker BioSpin, Billerica, MA, USA) as previously described [18].

### Neurobehavior

The CCI injuries for these studies were centered over the left frontal motor cortex. Consequently, neurobehavioral tests that primarily assessed motor and reflex functions were utilized for outcome assessments. Rats in the rMSC IV versus saline cohorts underwent neurobehavioral severity score-revised (NSS-R) evaluation at baseline and on days 1, 14, 28, and 56 after injury as previously described [9]. Rotarod testing was performed using a Med Associates rat rotarod (Med Associates, Inc., St. Albans, VT) [19, 20] in the rMSC IV versus saline cohorts on the same days as NSS-R testing. In each trial, the speed of rotation was increased from 0 revolutions per minute (rpm) to 35 rpm for a maximum of 10 minutes. Rats were tested in three trials per time point by rotarod, with recording of the mean duration on the device for each time point.

Motor coordination and limb function were tested through foot fault testing on a grid [21, 22] modified as follows and was performed on days 1, 14, and 28 after injury in IC rMSC and hMSC and their respective saline cohorts. Testing was not continued beyond 28 days because all injured cohorts were indistinguishable from baseline by 28 days. Foot fault testing was performed in an AccuScan Fusion Open Field Testing plexiglass box (16x16x16 inches) (Omnitech Electronics, Inc., Columbus, OH) into which a 1 x 2 inch wire mesh grid secured 1 inch above a touch sensor plate was inserted at the bottom of the enclosure. The rat was placed into the box and allowed to freely move atop the grid. A foot fault was scored if a paw slipped through the grid to trigger the touch sensor and counter. Testing sessions lasted for 5 minutes.

### Immunohistochemistry (IHC)

After euthanasia with pentobarbital, rats were perfused with ice-cold heparinized PBS, then 4% paraformaldehyde prior to brain extraction. Following equilibration in a sucrose gradient, brains were transferred to optimum cutting temperature (OCT) media and frozen in liquid nitrogen. Cryosections (10μm thick) were cut in the coronal plane, then mounted on glass slides and stored at -20°C. Representative sections underwent hematoxylin and eosin (H&E) staining using standard methods or Prussian blue staining as previously described [9].

Cytospin samples of cells prepared for injection and representative brain tissue sections from animals who received IC or IV MSC versus saline were stained using immunofluorescent staining techniques as previously described [9, 18]. For rMSC experiments, antibodies to stem cell markers CD44 (BD Pharmingen, San Jose, CA) and CD90 (Abcam, Cambridge, MA) were used. For hMSC experiments, the primary antibody used to detect hMSC was a mouse monoclonal antibody specific for human mitochondria (Abcam, Cambridge, MA). DAPI (4',6-diamidino-2-phenylindole) to mark cell nuclei was included with Prolong Antifade mounting media (Life Technologies, Grand Island, NY).

### Imaging Analysis

Cortical and lesion volumes were determined with MEDx software (Medical Numerics, Germantown, MD) as previously described [9]. Lesion volumes were demarcated by applying a segmentation algorithm (FAST—FMRIB Automated Segmentation Tool v3.1, University of Oxford, United Kingdom) to the T2-weighted MRI of CCI cortex. To compare changes in lesion volume of over time, absolute hemispheric and lesion volumes were converted to percent differences (% = [(Ipsilateral Cortex Volume—Lesion Volume)/(Contralateral Cortex Volume)] *100%).

### Statistical Analysis

For parametric data, one-way analysis of variance (ANOVA) and subsequent post-hoc Tukey was performed using Prism version 6.0d for Macintosh (GraphPad Software, San Diego, California, USA, www.graphpad.com), with a two-tailed *p* value of <0.05 considered significant. Nonparametric data (NSS-R) were analyzed by Kruskal-Wallis test for multiple comparisons in Prism, with a two-tailed *p* value of <0.05 considered significant. MRI data were reported as mean ± standard deviation (SD), while neurobehavioral data are reported as mean ± standard error of the mean (SEM), per conventions already established for data reporting in the imaging and neurobehavioral fields, respectively.

## Results

**Fig 2:**
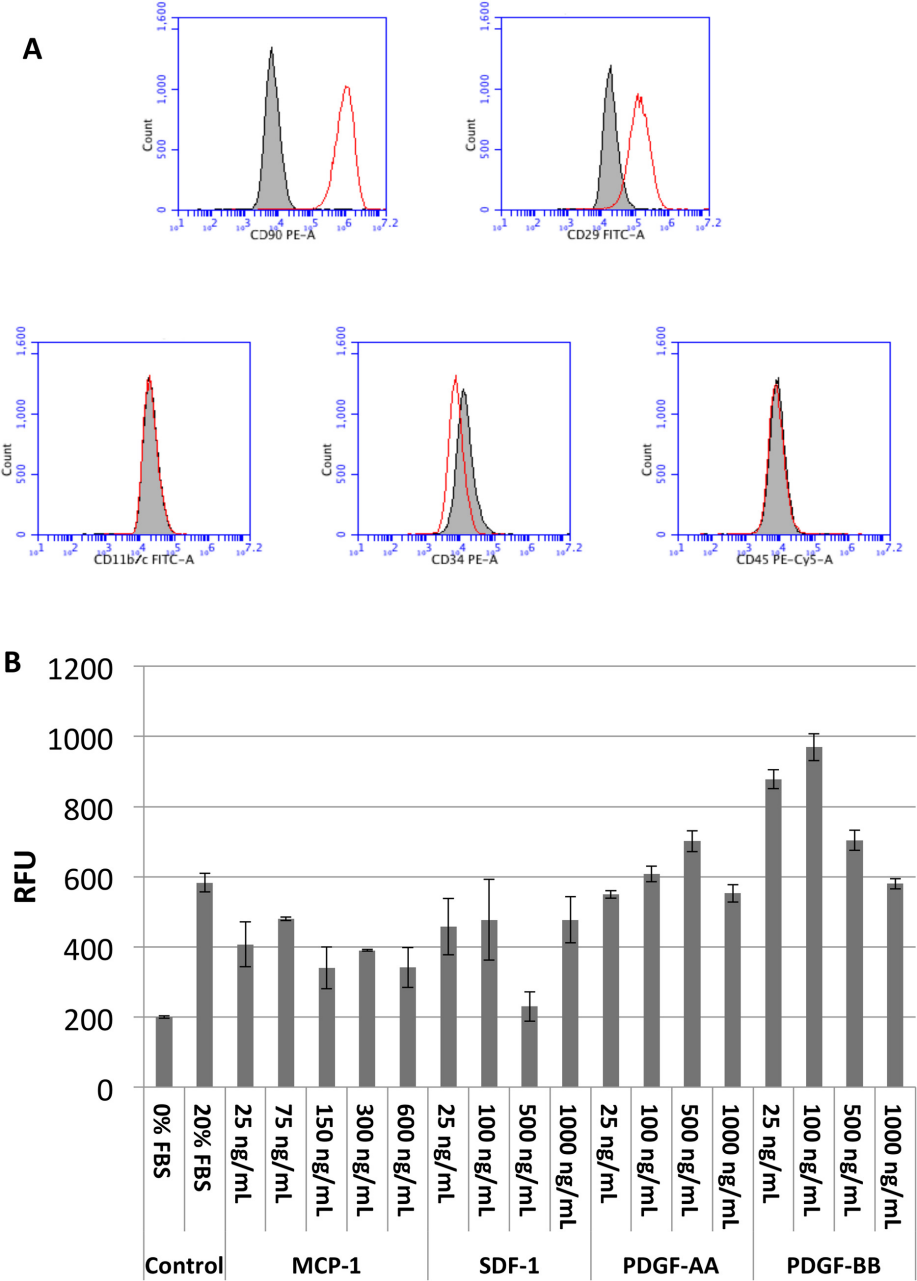
Characterization of rat mesenchymal stromal cells (rMSC). A) Flow cytometry for markers characteristic of rMSC. The dark shaded peaks represent the signal from isotype controls, while the clear shaded peaks indicate antibody staining. The rMSC isolated from bone marrow are positive for CD90 and CD29, but negative for CD11b7c, CD34, and CD45. B) Cell migration assay for rMSC, demonstrating chemotaxis of cells to serum and to factors known to cause chemotaxis of stem cells. RFU = relative fluorescent units; MCP-1 = monocyte chemoattractant protein-1; SDF-1 = stromal cell-derived factor 1; PDGF-AA = platelet-derived growth factor-AA; PDGF-BB = platelet-derived growth factor-BB.

The rMSC prepared for these studies demonstrated a typical expression pattern for rMSC by flow cytometry (Fig 2A), with strong staining for markers CD90 and CD29, and negative staining for CD11b, CD34, and CD45. Results of a typical rMSC migration assay are displayed in Fig 2B. The hMSC used in these studies were obtained after characterization by the NIH Bone Marrow Stromal Cell Transplant Center.

**Fig 3:**
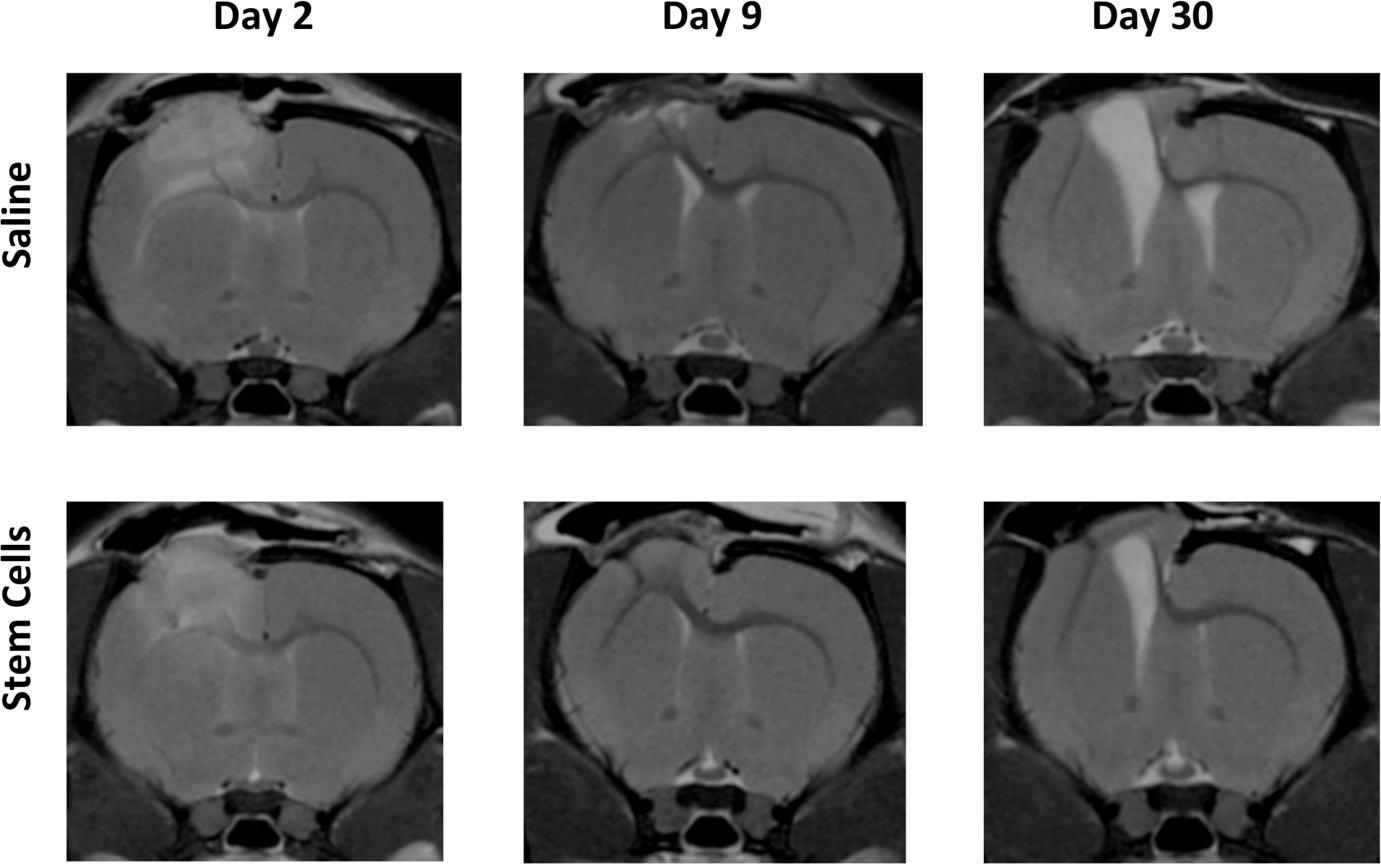
*In vivo* MRI tracking of CCI lesions over time. Representative MRIs at the same location for a rat treated with saline and a rat treated with rat mesenchymal stromal cells (rMSC) are displayed for days 2, 9, and 30.

**Fig 4:**
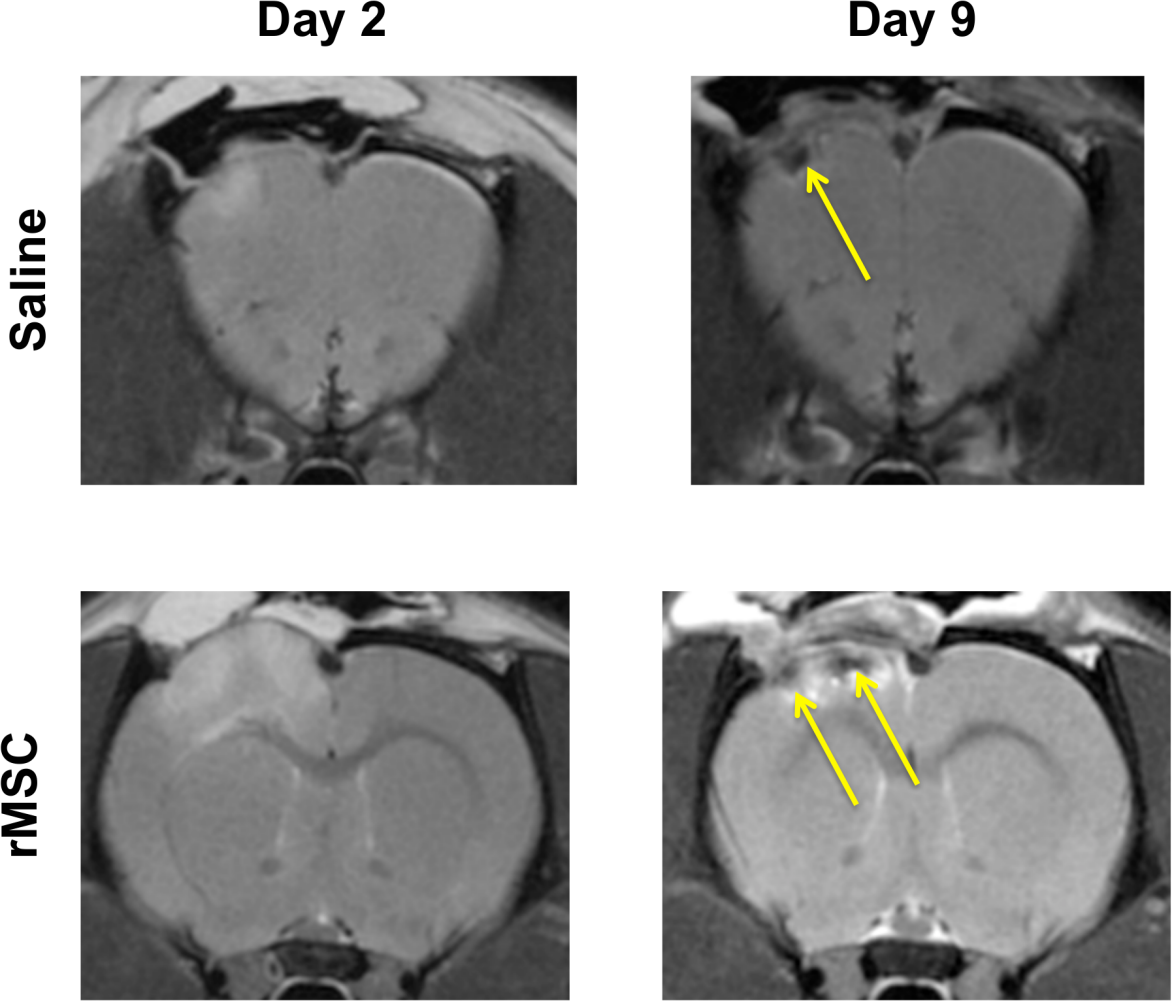
Evolution of hemorrhage post-injury. As previously reported in a parallel study [9], between days 2 and 9 post-CCI hemorrhage can evolve in the perilesion area. This has direct implications for *in vivo* cell tracking studies using SPION, since the MRI signals indicating hemorrhage and iron-labeled mesenchymal stromal cells cannot be distinguished. This may lead to misinterpretation of T2* hypointensities on MRI as evidence of labeled stem cell migration. The representative sections displayed here were selected to show how this evolving hemorrhage could be absent on Day 2, but present on Day 9. Because the development of hemorrhage is unpredictable, when it occurs, its precise location within the perilesion area varies from animal to animal. Arrows indicate hypointensities not present on Day 2 scans prior to cell injection that appeared by Day 9 and was due to the evolution of hemorrhage. rMSC = rat mesenchymal stromal cells.

**Fig 5:**
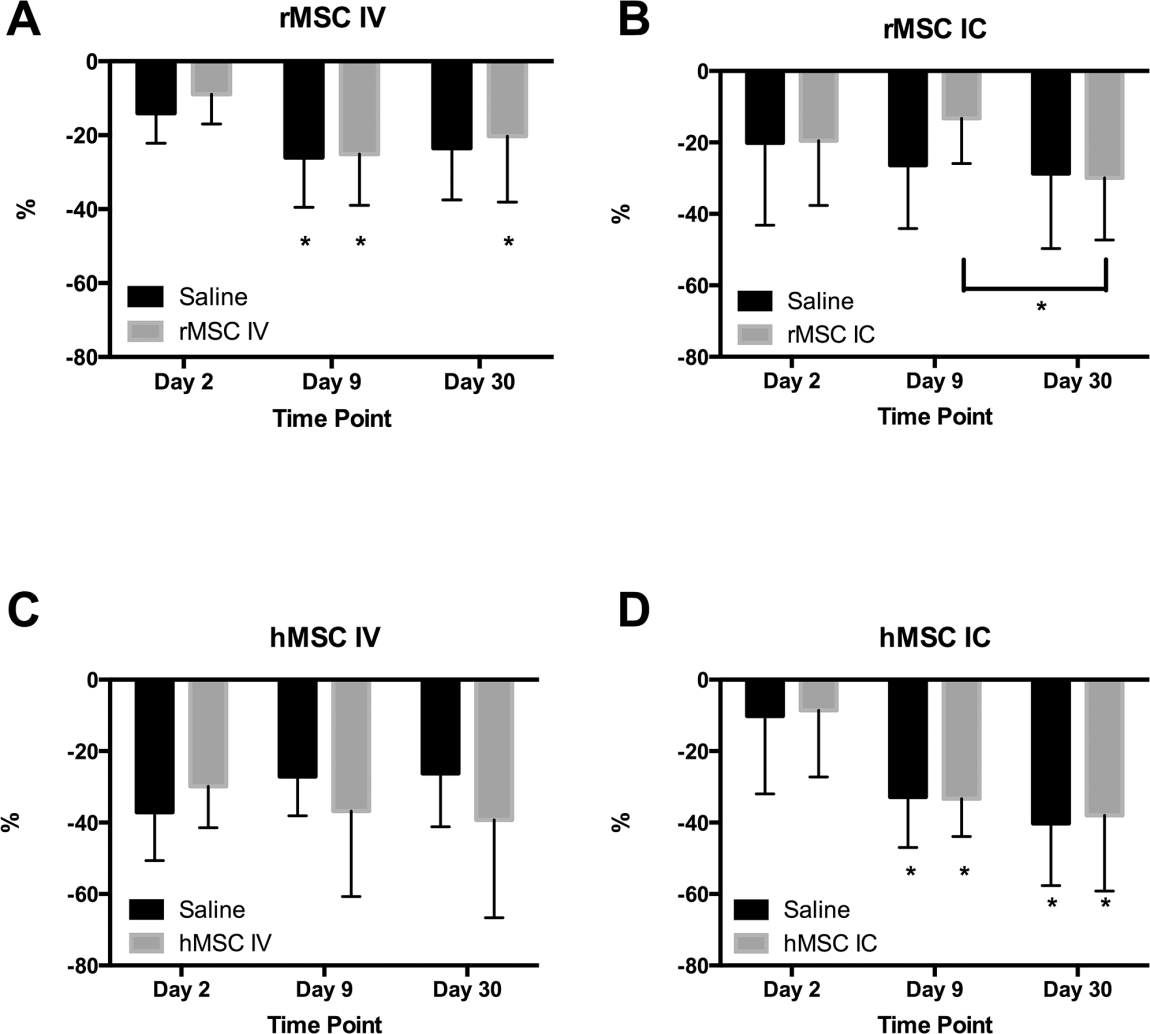
Analysis of *in vivo* MRI volumes after CCI over time. No differences were observed in MRI outcome parameters between saline and stem cell treated groups via either delivery method (intravenous (IV) or intracardiac (IC)) or type of cell (rat or human). A) rMSC IV; B) rMSC IC; C) hMSC IV; D) hMSC IC. % = [(ipsilateral volume—lesion volume)/contralateral volume) x 100%; rMSC = rat mesenchymal stromal cell; hMSC = human mesenchymal stromal cell; IV = intravenous; IC = intracardiac. * = p <0.05 relative to corresponding Day 2, except as noted for the rMSC IC, whether it references the difference between stem cell-treated Day 9 and Day 30.

The typical evolution of the CCI lesion over the time course of this study is depicted in Fig 3, with similar appearances noted on *in vivo* MRI for both saline and rMSC treated groups There was no difference in lesion appearance between the treatment cohorts of rats observed at 2 months post-injury by *ex vivo* MRI. *In vivo* tracking of SPION labeled rMSC by MRI was complicated by hemorrhage that could develop between Day 2 and Day 9 in both stem cell and saline-treated cohorts (Fig 4). Similar MRI results were observed with SPION labeled human MSC independent of the route of administration (data not shown). In all cohorts examined, there were no significant differences of MRI outcome measures between rMSC or hMSC treated cohorts and saline treated groups at any time point, regardless of whether cells were delivered via IV or IC injection. (Fig 5, Table 1).

**Table 1:** Efficacy of MSC versus saline treatments as assessed by MRI outcome.

		% Change = [(Ipsilateral Side-Lesion)/Contralateral Side)] x 100%				
		Day 2	Day 9	Day 30	ANOVA	ANOVA
Route	Treatment	Mean± SD	Mean± SD	Mean± SD	By Time	By Tx
IV	rMSC	-9±8	-25±14	-20±18	F = 12* p<0.001*	F = 0.5 p = 0.5
IV	saline	-14±8	-26±13	-23±14		
IC	rMSC	-19±18	-13±13	-30±17	F = 3* p = 0.04*	F = 0.5 p = 0.5
IC	saline	-20±23	-26±13	-29±21		
IV	hMSC	-30±12	-37±24	-39±27	F = 0.09 p = 0.9	F = 0.23 p = 0.6
IV	saline	-37±13	-27±11	-26±15		
IC	hMSC	-9±19	-33±11	-38±21	F = 23* p<0.0001*	F = 0.3 p = 0.6
IC	saline	-10±22	-33±14	-40±17		

IC = intracardiac, IV = intravenous; hMSC = human MSC; rMSC = rat MSC; SD = standard deviation; Tx = treatment

* = statistically significant interaction by two-way ANOVA

No statistically significant differences were observed by two-way ANOVA by treatment between MSC and saline cohorts at any time point. A statistically significant difference was observed by time between Day 9 or Day 30 versus Day 2 for all studies except the IV hMSC versus saline arms.

In the IV rMSC versus saline cohorts (Fig 5A), there were statistically significant differences in percent change of the ipsilateral side minus lesion volume versus contralateral side between days 9 and 30 versus Day 2. Furthermore, there were no statistically significant differences between rMSC and saline groups at any time point (IV Day 2: rMSC = -9±8%, saline = -14±8%; IV Day 9: rMSC = -25±14%, saline = -26±13%; IV Day 30: rMSC = -20±18%, saline = -23±14%; 2-Way ANOVA: by time point F = 12, p <0.001; by treatment F = 0.5, p = 0.5). In the IC rMSC versus saline cohorts (Fig 5B), there were no statistically significant differences in percentage change by treatment but there was by time point (IC Day 2: rMSC = -19±18%, saline = -20±23%; IC Day 9: rMSC = -13±13%, saline = -26±18%; IC Day 30: rMSC = -30±17%, saline = -29±21%; 2-Way ANOVA: by time point F = 3, p = 0.04; by treatment F = 0.5, p = 0.5).

After administration of hMSC IV injections (Fig 5C), there were no significant differences in percentage change by either treatment or time (IV Day 2: hMSC = -9±19%, saline = -10±22%; IV Day 9: hMSC = -33±11%, saline = -33±14%; IV Day 30: hMSC = -38±21%, saline = -40±17%; 2-Way ANOVA: by time point F = 0.09, p = 0.9; by treatment F = 0.23, p = 0.6). With hMSC IC injections (Fig 5D), there was a significant difference by time point for days 9 and 30 relative to Day 2, but no difference by treatment groups (IC Day 2: hMSC = -30±12%, saline = -37±13%; IC Day 9: hMSC = -37±24%, saline = -27±11%; IC Day 30: hMSC = -39±27%, saline = -26±15%; 2-Way ANOVA: by time point F = 23, p <0.0001; by treatment F = 0.3, p = 0.6).

**Fig 6:**
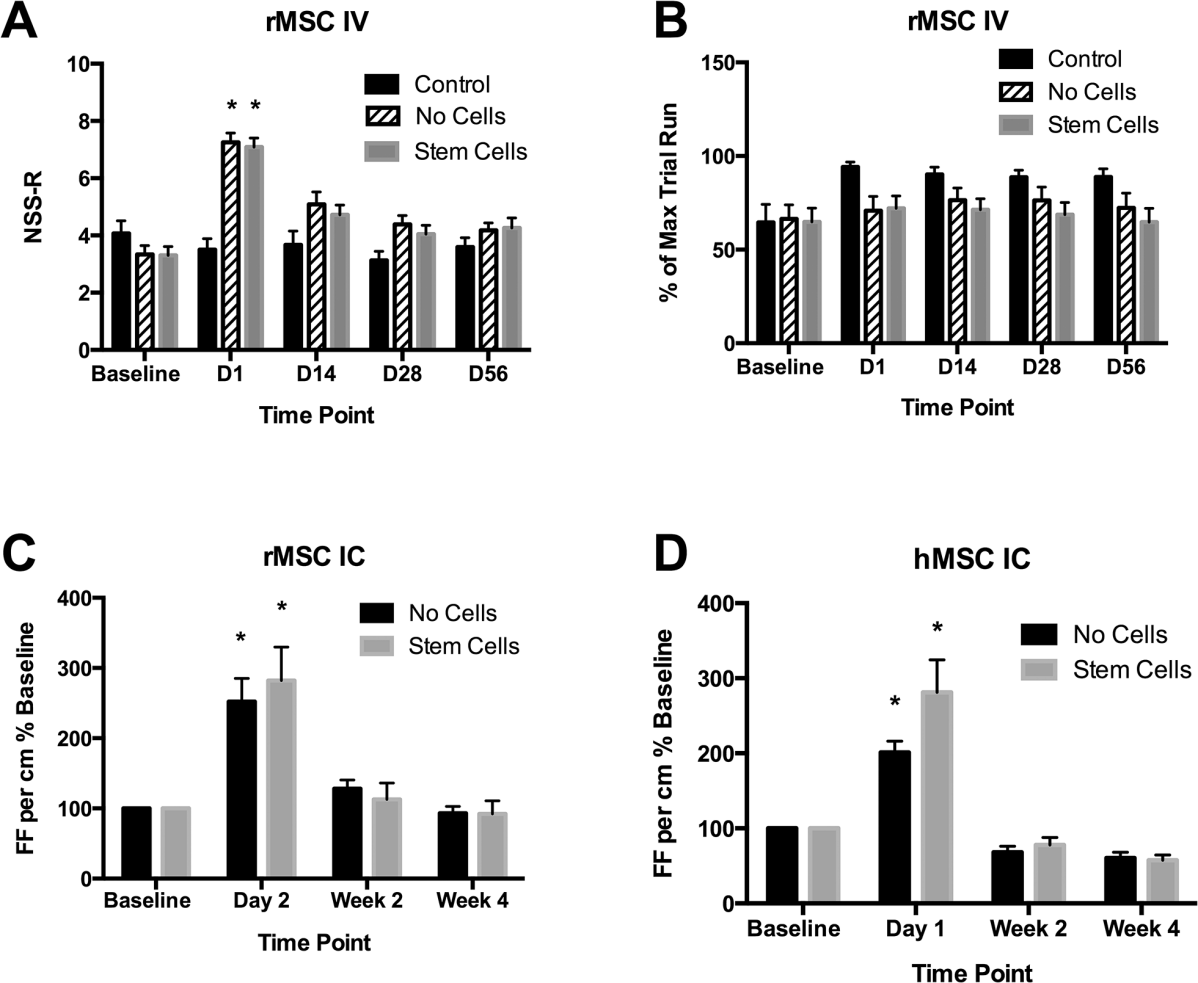
Neurobehavioral analyses after focal contusion in the rat. A) While differences in neurobehavioral severity score-revised (NSS-R) testing were seen between injured rats and both control and baseline at Day 1 post-injury, no treatment effect was observed at any time point in the intravenous rat mesenchymal stem cell (rMSC) cohorts. B) Rotarod testing also failed to distinguish between saline and rMSC treatment groups over the time course. C) Foot fault analyses found difference on Day 2 in the rMSC intracardiac cohort, but only in comparison to baseline. D) Foot fault analysis in the human mesenchymal stem cell (hMSC) intracardiac cohort similarly showed differences early after injury, which normalized over time. * = p < 0.05.

Neurobehavioral analysis in the IV cohorts of rats included NSS-R scoring and rotarod testing (Fig 6A and 6B). By NSS-R, significant differences were seen between all baseline groups and the MSC- and saline- treated groups (NSS-R: baseline control = 4.1±0.4; baseline saline-treated = 3.3±0.3; baseline MSC-treated = 3.3±0.3; Day 1 control = 3.5±0.4; Day 1 saline-treated = 7.3±0.3; Day 1 MSC-treated = 7.1±0.3; p<0.0001). These differences in baseline and Day 1 post-injury NSS-R scores, where higher scores indicate worse functioning, demonstrates that the injury produced neurobehavioral changes. No significant differences were observed at any time point between saline-treated and MSC-treated groups, however, indicating that there were no differences in neurobehavior scores that could be attributed to treatment group. For rotarod testing, no significant differences were observed for the percentage of maximum trial run between saline and rMSC-treated groups at any time point (Fig 6B).

For IC cohorts, behavioral testing was performed by foot fault analysis since no differences were observed with rotarod between saline treated and either IV MSC treated cohorts. No significant differences were seen between saline and either MSC-treated groups at any time point (Fig 6C and 6D). The only significant differences for foot fault scores were seen between Day 1 or 2 and other time points, including baseline (Fig 6C: Day 2 saline-treated = 2.5±0.4; Day 2 rMSC-treated = 2.8±0.5; Week 4 saline-treated = 0.9±0.1; Week 4 rMSC-treated = 0.9±0.2, p<0.05; Fig 6D: Day 1 saline-treated = 2.0±0.2; Day 1 hMSC-treated = 2.8±0.4; Week 4 saline-treated = 0.6±0.1; Week 4 hMSC-treated = 0.6±0.1, p<0.05).

**Fig 7:**
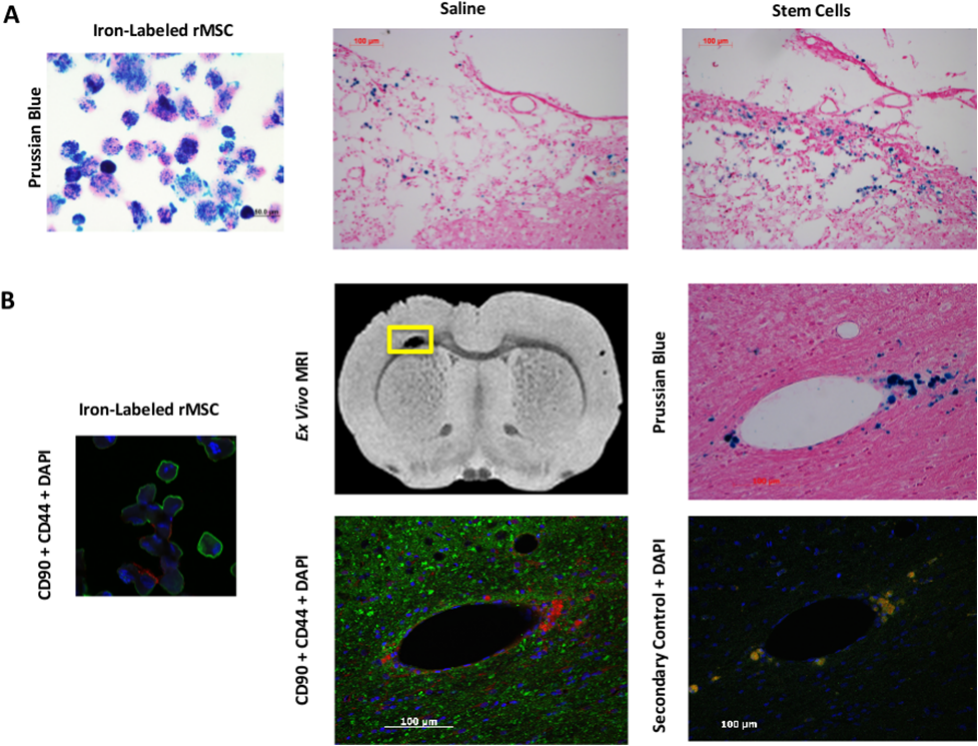
Immunohistochemical and immunofluorescent analyses of brains after stem cell treatment. The images shown are from rats in the saline versus rMSC cohort via intravenous dosing, although similar data are seen after intracardiac injections. A) Staining of a cytospin of iron-labeled rMSC and sections of injured cortex from saline and stem cell treated rats at 10 days post-injury demonstrates Prussian blue staining in the iron-labeled rMSC used for injection and in both saline and stem cell-treated animals. In conjunction with MRI, most of this staining appears to be secondary to hemorrhage. Macrophages have likely ingested the hemosiderin, resulting in their staining positive for Prussian blue. Similar results were observed with hMSC (data not shown). B) A very small subset of cells in these Prussian blue positive staining areas show dual-labeled immunofluorescence to stem cell markers CD90 (green) and CD44 (red), suggesting, but not proving, that these few cells may be labeled MSC that have migrated into the brain. CD90 is also a neuronal marker in rats. The yellow box on the *ex vivo* MRI section from a stem cell-treated rat at 10 days post-injury shows the area at the junction of ipsilateral cortex and corpus callosum subsequently examined by immunofluorescence. The secondary control section demonstrates background immunofluorescence obtained by incubation with the secondary antibodies alone in the absence of prior primary antibodies. In saline versus hMSC treatment cohorts, limited staining of cells with anti-human mitochondrial antibody was also observed (data not shown). Scale bars are 50 μm in the images of cells alone, and 100 μm for the tissue sections. DAPI = 4',6-diamidino-2-phenylindole appears blue in fluorescent staining.

Regardless of route of injection, transplanted rat or human MSCs were undetectable by dual Prussian blue and immunofluorescent staining at late time points (4 weeks and 2 months; 0% of injected MSC; data not shown). Separate cohorts of animals were given IV or IC injections of rMSC or hMSC with the dosing regimens reported in Fig 1, then harvested 1 to 3 days after the last injection, to see if transplanted stem cells could be detected sooner after injection. The greatest proportions of cells were found trapped in lung and spleens (data not shown). Small numbers of Prussian blue stained cells were detected in the injured cortex of animals at Day 10 post-injury after IV administration of rMSC (Fig 7A), but Prussian blue stained cells were also observed in the saline-treated cohort. In the rMSC-treated group a small subset of these Prussian blue positive cells stained positively for rMSC markers CD90 and CD44 at Day 10 post-injury (Fig 7B)(less than 0.0005% of the injected number of MSC), versus none in the saline-treated cohort. Likewise, few hMSCs were detected in the brain post-injury by anti-human mitochondrial antibody staining in hMSC but not saline-treated cohorts (data not shown).

## Discussion

The major findings of this study were as follows: 1) administration of three IV doses of either rat or human MSC on days 3, 5, and 7 after CCI does not improve MRI or neurobehavioral outcomes over a 1–2 month time course in the female rat; 2) administration of two IC doses of either rat or human MSC on days 0 and 1 after CCI fails to improve outcomes; 3) the evolution of hemorrhage in lesion and perilesion areas between days 2 and 9 post-CCI complicates interpretation of *in vivo* MRI tracking of SPION-labeled stem cells; and 4) rat or human MSC were not detectable in CCI-injured brains at 2 months post-injury when administered by IV or IC routes.

In these investigations, the effects of IV MSC delivered on days 3, 5, and 7 post-TBI were examined over a 1 month time course for *in vivo* MRI and continued to a 2 month time course post injury for neurobehavioral and *ex vivo* analysis. Our initial experimental design was based upon the published literature [6–8] that had reported neurobehavioral improvements after TBI after single intravascular doses of MSC. While at early neurobehavioral time points differences were observed between injured and control cohorts, there were no significant differences seen between rat or human MSC-treated and saline-treated groups over the time course (Fig 6). Likewise, by MRI outcomes, there were no statistically significant differences between MSC-treated and saline-treated groups at days 2, 9, or 30 (Fig 5). Similarly, no improvement in outcomes by MRI or neurobehavioral outcomes was seen with IC administration of MSC on days 0 and 1 after injury (Figs 5 and 6). This lack of differences was consistent whether MSC were derived from rats or humans. Our cells displayed characteristic behaviors of rMSC (Fig 2), including expression of appropriate cell surface markers and migration to appropriate chemotactic agents MCP-1, SDF-1, PDGF-AA, and PDGF-BB [23, 24], with the highest levels of migration seen in conjunction with PDGF-BB.

In our studies, we used MSC labeled with SPION for MRI tracking purposes. In the IV cohorts, initial MRI scans were performed on Day 2 post-injury, prior to the administration of stem cells on days 3, 5, and 7. The first follow up scans were done on Day 9. We found in a parallel study [9] that injured rats without evidence of hemorrhage at Day 2 can develop hemorrhage between days 2 and 9 (Fig 4). Both hemorrhage and SPION have similar MRI signals (both are paramagnetic and appear hypointense on T2-weighted images), complicating the interpretation of efforts to track stem cell by *in vivo* and *ex vivo* MRI [25–27]. Post-mortem histological methods to distinguish between hemorrhage and SPION-labeled cells are also difficult, because both stain with Prussian blue.

No long-term differences between control rats and treatment groups (MSC or saline) were observed by NSS-R or rotarod testing in the IV cohorts (Fig 6A and 6B). Therefore we piloted a foot fault analysis protocol based on a previous report [21] that was proposed to be more sensitive to detecting differences in motor function. No differences were observed in the IC MSC treatment groups compared to saline controls, and the performance of injured animals was indistinguishable from controls by 4 weeks post-injury (Fig 6C and 6D). Short-term differences were observed between injured versus control cohorts via all neurobehavioral assessments, indicating that the NSS-R and foot fault analyses were both sensitive enough to detect functional changes. However, at no other time points were differences noted between MSC versus saline treatment groups, confirming the lack of a treatment effect. The long-term normalization of behavior between injured and non-injured cohorts also indicates that the rat’s intrinsic recovery from injury was robust enough to mask differences by these neurobehavioral measurements.

The failure of IV or IC MSC to influence neurobehavioral outcomes in the present study is in distinct contrast to several earlier studies investigating the efficacy of MSC after focal contusion injury in the rat [6–8, 28–32] but consistent with another report [33] (Table 2). Assessment of cell localization in the initial studies in male rats occurred via bromodeoxyuridine (BrdU) staining [7, 8], which subsequent investigations have found is not necessarily indicative of the presence of live MSC [11, 34]. These early studies all reported significant numbers of MSC reaching the damaged brain itself, ranging from 14–19% at 7 days after intra-arterial injection [6] to 0.46 to 2.2% at 14 days after intravenous injection [7, 8, 29, 35]. At 3 months post-injury and transplantation, many fewer MSC were reported to be present in either male or female rats, although improved neurobehavioral scores were still reported after IV injections of 4 or 6 x 10^6^ cells [31, 32].

**Table 2:** Comparison of experimental studies of mesenchymal stromal cells administered after focal contusion TBI in the rat.

Study	Strain	Sex	Injury	Cells ^1^	Route	Cell Dose	Dose Time	Endpoint	Result
[6]	W	M	CCI LCx B/L Cr	rMSC (M)	ICA	10^6^	24 hours	7 DPI	+ BrdU
[7]	W	M	CCI LCx B/L Cr	rMSC (M)	IV	2 x 10^6^	24 hours	14 DPI	+ NB; + BrdU
[8]	W	F	CCI LCx B/L Cr	rMSC (M)	IV	2 x 10^6^	24 hours	7 or 14 DPI	+ NB
[36]	W	M	CCI LCx B/L Cr	rMSC	ICR	1 x 10^6^	24 hours	8 DPI	+ NB
[35]	W	M	CCI LCx B/L Cr	hUCB	IV	2 x 10^6^	24 hours	28 DPI	+ NB
[30]	W	M	CCI LCx B/L Cr	hMSC	IV	1 to 2 x 10^6^	24 hours	1 month	+ NB
[29]	W	M	CCI LCx B/L Cr	rMSC (M)	IV	2 x 10^6^	24 hours	15 DPI	+ NB
[31]	W	M	CCI LCx B/L Cr	hMSC	IV	(2, 4, or 8) x 10^6^	24 hours	3 months	+ NB
[32]	W	F	CCI LCx B/L Cr	rMSC (M)	IV	(2, 4, or 8) x 10^6^	7 days	3 months	+ NB
[37]	W	F	Feeney RCx	rMSC (M)	ICR	5 x 10^6^	2 months	4 months	+ NB
[33]	SD	M	CCI RCx	rMSC	IV	4 x 10^6^	24 hours	2 or 14 DPI	no effect
[38]	SD	M	CCI RCx	hMSC	IVC	20 capsules of 3200 cells	Before injury	14 DPI	decreased neuronal loss
[39]	SD	F	Feeney RCx	hMSC	IV or ICA	0.5 or 2.5 x 10^6^	24 hours	1 to 5 DPI	+ HuN
[40]	SD	M	Feeney LCx	rMSC	ICR	0.5 x 10^6^	24 hours	3 weeks	+ SPION label
[41]	W	F	Feeney RCx Mod v. Sev	rMSC (M)	ICR	5 x 10^6^	2 months	4 months	+ NB (Mod)
[42]	W	F	Feeney RCx	rMSC (M)	IV	15 x 10^6^	2 months	4 months	no effect
[43]	SD	M	Feeney RCx	AMNC	ICR	1 x 10^6^	24 hours	28 DPI	+ NB
[44]	SD	M	Freeze RCx	rMSC	ICA	2 x 10^6^	7 days	5 weeks	+ NB
[45]	SD	M	CCI RCx	MAPC	IV	2 x 10^6^/kg or 10 x 10^6^/kg	2 and 24 hours	120 DPI	+ NB
[46]	SD	M	CCI RCx	AMNC	IV	2 x 10^6^/kg	74 hours	4 Weeks	+ NB
[47]	SD	M	CCI RCx	hUCB	IV	4 x 10^6^	7 days	56 DPI	+ NB
This study	W	F	CCI LCx	rMSC (M) or hMSC	IV	5 x 10^6^	3, 5, 7 days	56 DPI	no effect
					IC	1 x 10^6^	0, 1 days	30 DPI	no effect

^**1**^ The sex of origin of the stem cells is listed in parentheses when available in the published literature.

AMNC = autologous bone marrow-derived mononuclear cells; B/L Cr = bilateral craniectomies; + BrdU = positive staining for 5-Bromo-2’-Deoxyuridine; CCI = controlled cortical impact model; DPI = days post-injury; Feeney = Feeney weight-drop open skull contusion model; F = female; + HuN = positive antibody staining for human nuclear antigen; hMSC = human MSC; hUCB = human umbilical cord blood; IC = intracardiac; IV = intravenous (either jugular or tail vein); ICR = intracranial; ICV = intracerebroventricular; LCx = left cortex; M = male; MAPC = multipotent adult progenitor cells; + NB = improvement in neurobehavioral outcome; rMSC = rat MSC; RCx = right cortex; SD = Sprague-Dawley; + SPION = + for superparamagnetic iron oxides staining; W = Wistar

In contrast, Harting et al [33] injected 4 x 10^6^ rMSC at 24 hours after stroke injury by tail vein, yet found that only 0.0005% of the cells (fluorescently tagged prior to injection) reached the brain in a rat TBI model. The vast majority of cells were trapped in the lungs [33, 48]. By 14 days post-injury, very few cells could be found anywhere in the brain, and no neurobehavioral differences were observed between treated and untreated groups [33]. Investigations of MSC homing in other disease models also report that after intravenous injection MSC get trapped in peripheral organs such as the lung [49, 50] and the few that get incorporated into the brain do not persist long-term [51]. This failure of MSC to either enter the brain via peripheral injection routes or to persist long-term is consistent with our findings in the current study. Of note, at the time these studies were performed, detachment of MSC grown in culture was performed with trypsin. Recently, it has been found that pronase treatment of MSC to detach cells from the culture results in less cell trapping in the lungs [52].

MSC have also been directly administered into the brain in several studies using a different model of TBI, a weight drop model. When MSC were transplanted intracerebrally at 2 months after injury in adult male Wistar rats, treated animals with moderate but not severe TBI demonstrated functional improvement by 4 months post-injury [37, 41]. In contrast, delaying IV administration of MSC until 2 months post-injury had no benefit on neurological outcomes or histopathological analyses [42]. Osanai et al. [44] reported functional recovery and persistence of PKH26 and quantum dot-labeled MSC in the perilesional area up to 28 days after TBI induced by a cortical freezing injury. These authors administered fluorescently-labeled MSC to male rats via intraarterial injection in the ipsilateral carotid artery 7 days after TBI. Direct transplantation of autologous MSC into the brain of male rats at 24 hours after CCI also resulted in neuroprotection and angiogenesis [43], demonstrating the possible advantage of this approach for delivering cells albeit not via an approach that is clinically relevant for most TBI patients.

A major advantage of our study is that we had larger numbers of animals per cohort than in prior investigations of the efficacy of MSC after TBI. Our MRI studies had cohorts of 12 to 17 rats per arm, consistent with a sample size that would result in a power of 0.8 and alpha of 0.05 [9], compared with other studies touting the value of cell therapy in which the results from 4 rats per arm were used in the earliest studies of MSC after CCI [6–8]. Given the inherent variability in the CCI model [9], cohorts of less than 10 rats per treatment arm may result in underpowered analyses. We also investigated both rat and human MSC in these studies, administered by two different routes (IV versus IC) and demonstrated no neuroprotective or behavioral effects compared to saline controls. The profile of administered cells in the current study is similar to other reports [53, 54].

The present study aimed to investigate whether multiple intravascular doses of MSC in the subacute phase after injury would improve outcomes by MRI and neurobehavioral outcomes. Similar doses were used as in the original single dose MSC studies [6–8] (Table 2), yet no differences were seen by either outcome measure between treated and untreated cohorts. This contrasts with results seen in early trials of MSC after TBI, but not of subsequent studies by other investigators [6–8, 33].

Differences in techniques may contribute to the variation in efficacy of MSC. In the original TBI studies with MSC, the investigators did bilateral decompressive craniectomies prior to TBI, but only performed CCI over the left motor cortex [6–8, 29], while in the current study and others [33, 45, 46] a single craniectomy was performed though which the injury occurred. Data from a recent clinical trial suggests that patients receiving bilateral decompressive craniectomies post-TBI have worse long-term outcomes than those with single craniotomies [55], suggesting the possibility that bilateral versus single craniotomy injury models may lead to different severities or consequences. Different locations of injury may also account for discrepancies, and/or different severities of injury. In addition, the specific neurobehavioral tests used to assess outcomes differ among papers published in the literature, which makes comparison across studies difficult and challenging to replicate, as do comparison of short-term versus long-term studies [56]. The passage number of MSC used may also affect efficacy of treatment. The first reports reporting benefit of MSC after experimental TBI used cells after one passage [7, 8], earlier than in the present study and others [33]. After a single passage, MSC cultures contain a mixed population of cells, while later passage numbers result in a more homogeneous population of MSC, suggesting that some of the differences in efficacy between studies may be due to different populations of cells administered [33].

Whether the contradictory reports of transplanted cells reaching the brain and surviving is due to labeling techniques used, differences in mechanism of injury, timing of delivery, or other factors remains unclear. The early stem cell studies may also have overestimated the migration of stem cells into the perilesion area. Assessment of cell localization in the initial studies in male rats occurred via BrdU staining [7, 8], which subsequent investigations have found is not necessarily indicative of the presence of live MSC [11, 34]. BrdU labeling can be transferred from pre-labeled donor cells (living or dead) to host cells [34]. Other intracellular labels such as SPION and GFP can also be transferred from labeled MSC to resident tissue macrophages [11]. We have observed a robust macrophage response that occurs in the first week after CCI injury that may also complicate interpretation of short-term studies of labeled stem cell survival after TBI [18].

In addition, confirmation with fluorescent labels or antibody staining can be complicated by the presence of autofluorescence of cells post TBI. We observed autofluorescence in the hemorrhagic TBI lesions in our prior studies [9] and this finding has additional implications for cell tracking studies. Intact red blood cells are autofluorescent, a property that has been used to detect microhemorrhages in post-mortem studies [57]. The striking extracellular autofluorescence in necrotic hemorrhagic areas of the brain is attributed to a clotted chromolipid called hemoceroid, which may eventually be ingested by macrophages [58]. Techniques to control this background autofluorescence are limited and imperfect [59]. The prevalence of autofluorescence cells and extracellular material in areas of hemorrhagic injured brain may lead to the misinterpretation of the presence of SPION-labeled cells unless the appropriate controls are performed. In the current study, immunofluorescent control stains were performed for all brains, in which background immunofluorescence was documented in the absence of primary antibody.

Another factor that may introduce variability among studies is biological sex, of both MSC donors and of recipients. The majority of stem cell transplantation experiments have occurred in male rats, with only a small subset of studies done in female rats (Table 2). Half of the human population is female, and both sexes are at risk for traumatic brain injury. Female rats were used as MSC recipients for the present study for two reasons. Female rats grow more slowly than males, and there was a limit on the size of the rats we could fit into the MRI coil that was available at the time of these studies. The other reason we used female rats was because limited numbers of TBI experiments reported in the literature were done in female rats, and there is growing recognition of the importance of investigating both sexes in biomedical research [60]. We thought it important to ascertain in a large sample size if female rats behaved the same as males with respect to peripheral administration of MSC after TBI.

In contrast to the initial reports which found that IV administration of MSC from male donor rats were efficacious in both males [7] and females [8], we found no effect of multiple doses of IV or IC MSC after CCI in female rats. Either intrinsic sex differences or variations in ovarian hormones through the female rat’s cycling may contribute to both a) increased variability in lesions as well as b) differences in responses to stem cell treatment. The sex of derivation of the original stem cells may also play a role in efficacy of transplantation [61], although data for solid tissue donation is mixed with no effect in lung [62] and worse outcomes for kidney transplants from female donors to male recipients [63, 64]. Whether there are sex-of-origin of recipient or donor effects related to MSC transplantation after experimental TBI is unclear at the present time and requires further investigation.

While data are conflicting as to the efficacy of peripherally administered MSC, treatment with multipotent adult progenitor cells (MAPC) cells demonstrates more promise in preclinical studies [45, 46, 65, 66]. It has been hypothesized that the effects of these peripherally administered MAPC in TBI may be predominantly mediated by peripheral interactions of stem cells with splenocytes, perhaps by altering the ratio of proinflammatory versus anti-inflammatory macrophages [65]. Treatment of rats post-CCI with MAPC (which are smaller diameter, more primitive cells in the lineage of bone-marrow derived MSC), resulted in preserved BBB integrity and increased numbers of CD4+ splenocytes and levels of IL-4 and IL-10 globally versus untreated injured animals. Intravenous delivery of MAPC post-CCI also triggers increased T regulatory cells in the spleen and plasma and an increased M2/M1 brain macrophage ratio in mice [66]. Improvements in spatial learning and reduced neuroinflammation post-CCI were observed in male rats after MAPC administration [45], and after autologous bone marrow-derived mononuclear cells (AMNCs) [46].

The AMNC approach may be clinically preferable, as it permits a) transfusion of the patient’s own cells after enrichment in a b) timeframe that is clinically relevant (2–3 days after injury), with minimal time for isolation and reinjection (2 hours) [46, 67]. Clinical Phase 1 trials investigating AMNC after brain injury have demonstrated that AMNC isolation and intravenous re-administration is safe in children within 48 hours after severe TBI (ClinicalTrials.gov #NCT00254722) [68] and in adults within 72 hours after acute ischemic stroke (ClinicalTrials.gov #NCT00859014) [69].

In addition to the points discussed above, there are other limitations to the current study that employed two different methods of MSC delivery at multiple time points. The IC studies delivered MSC in the acute time frame (right after injury and on Day 1), at the height of injury, while the IV studies administered MSC at three sub-acute time points (days 3, 5, and 7). MSC administered acutely or subacutely did not persist as long-term transplants. Changes in the inflammatory milieu of the injured brain within the first week after injury [18] may contribute to the different outcomes of labeled MSC given via IC injection acutely or IV injection within the first 7 days in in the present study in contrast to the persistence of labeled MSC transplants administered by intracarotid injection at 1 week after injury [44]. A comprehensive analysis of delivery of multiple doses of MSC from different sources delivered at acute, subacute, versus chronic time points was beyond the scope of this report. Another limitation of this study is that only non-autologous or xenogenic adult bone marrow derived MSC were used and were found to be ineffective in altering clinical or pathological outcomes. Whether different stromal cells or potency of MSC [70] derived from a different population would be efficacious is unknown.

The experiments described here were originally designed to test the hypothesis that multiple doses of bone marrow derived mesenchymal stromal cells would be more efficacious than a single early dose. We chose a peripheral vascular approach because we were trying to find a method of administration that would be applicable to a broader clinical population than direct intracerebral injection. We based our initial experimental design upon the early successful reports of stem cell administration, where cells were given via multiple different routes (IV, ICA, and directly into the brain, from both rat and human) at a variety of time points from 24 hours to 7 days in either male or female rats, all of which were reported to be successful, at least by neurobehavioral outcomes (Table 2). We used mesenchymal stromal cells derived in a similar manner those described in the original publications (a slightly higher passage number had to be used because of the number of doses needed). We chose initial neurobehavioral testing (Neurological Severity Score and rotarod testing) similar to what those original authors had reported to be successful [6–8], concentrating upon tests of motor function because our injury was centered over the left frontal motor cortex, with little damage to the hippocampus noted on MRI or histopathology. Despite our efforts, in this population of female Wistar rats we were unable to replicate the earlier reported successes via either intravenous or intracardiac dosing.

In conclusion, the present study fails to replicate the early reports of cell homing and improved neurobehavioral outcomes after peripheral vascular administration of MSC. No difference in MRI outcome measures or neurobehavioral outcomes was observed among the various treatment groups. We conclude that non-autologous MSC infusions via peripheral vascular routes of administration are not a promising avenue for stem cell therapy after TBI in all populations. Initial studies by other laboratories [43, 46, 67–69] suggest that autologous cells, such as AMNCs, that can be quickly isolated and administered back to the same host, warrant further investigation.
